# Civil liability of the ophthalmologist in the São Paulo Court of Appeals

**DOI:** 10.1590/S1679-45082017AO3781

**Published:** 2017

**Authors:** Isabel de Fátima Alvim Braga, Kelly de Oliveira Vieira, Thiago Gonçalves dos Santos Martins

**Affiliations:** 1 Fundação Oswaldo Cruz, Rio de Janeiro, RJ, Brazil.; 2 Universidade Federal do Estado do Rio de Janeiro, Rio de Janeiro, RJ, Brazil.; 3 Universidade Federal de São Paulo, São Paulo, SP, Brazil.

**Keywords:** Damage liability, Liability, legal, Ophthalmology/legislation & jurisprudence

## Abstract

**Objective:**

To conduct a data survey on the subject of medical claims involving civil liability in ophthalmology at the São Paulo Court of Appeals.

**Methods:**

A case law research was carried out on the São Paulo Court of Appeals website searching for the keyword “ophthalmologist” for all years until 2016.

**Results:**

Of the 65 cases found, 29 were selected. There has been an increase in the number of claims in ophthalmology, especially in surgical procedures on the anterior chamber of the eye. Most lower court judgments were for defendant.

**Conclusion:**

The study suggested the need for specialists to exercise the required amount of care when treating the patients, so that they may understand the risks inherent to the procedure. Despite the increase in claims, most decisions were favorable for the physician.

## RESUMO

**Objetivo:**

Realizar um levantamento de dados sobre a temática dos processos envolvendo responsabilidade civil em sede da Oftalmologia no Tribunal de Justiça do Estado de São Paulo.

**Métodos:**

Foi realizada uma pesquisa jurisprudencial por palavra-chave no site do Tribunal de Justiça do Estado de São Paulo com o seguinte termo de pesquisa “oftalmologista” para todos os anos até 2016.

**Resultados:**

Foram encontrados 65 processos, dos quais 29 foram selecionados. Observou-se tendência ao aumento de processos na área, e predominância de processos em procedimentos oftalmológicos cirúrgicos em câmara anterior do olho. A maioria das sentenças de primeira instância foi improcedente.

**Conclusão:**

O estudo aponta para a necessidade de atenção dos especialistas para com o paciente, no sentido de que ele compreenda os riscos inerentes ao procedimento. Apesar do aumento de processos, a maioria permanece favorável ao médico.

## INTRODUCTION

Despite advances in all medical areas, an ancient problem still makes healthcare professionals feel not confident: medical malpractice, which may cause losses both to patients and their families, prolonging the length of hospital stay and considerably increasing hospital costs. Additionally, said errors directly affect the live of devoted healthcare professionals committed to their patients’ well-being. Today, as medical malpractice has become increasingly important, it is a recurrent topic in media hype.^1^

In the Code of Hammurabi, from the First Babylon Empire, dating back to the 18^th^ century BC, there were rules that punished physicians for their errors.^1^ Despite the evolution of laws, the practice of medicine has become a risky activity in our country.

Physicians may stand trial by two courts: the court of general jurisdiction that follows the precepts of the Criminal and Civil Codes and the Medical Boards, whose decisions are based on the Code of Medical Ethics.^2^ The civil proceeding aims to make whole for pecuniary damage and pain and suffering; the criminal proceeding, for protecting the society; and the ethical proceeding filed with the Regional Medical Board aims at disciplining professional medical conduct. The most important article of the Code of Medical Ethics that characterizes malpractice is article 29, which provides that “physicians are prohibited from practicing harmful professional acts to the patient, which may be characterized malpractice, recklessness or negligence”.^2^

Medical civil liability results from fault in the broad sense, which includes intent, that is the willful determination to cause damage and fault in the strict sense.^3^ If damage is proved, the causal relation is to be checked to establish if the injury was actually caused by either the professional’s action or omission.

There are three forms of fault: recklessness (act without the due care), negligence (act of omission) and malpractice (lack of skill). The courts understand that in the doctor-patient relationship an agreement is executed between them for the rendering of services, despite unwritten.

Physicians are bound by no result obligation and the obligation of making all resources available to achieve the best possible result. If the expected result is not reached -- and in case there is no negligence, malpractice or recklessness – there will be no fault, in principle, which would lead to the payment of damages in the civil level.

However, for professionals bound by result obligation – if the expected result is not reached, regardless of the existence of fault – the agreement is breached and the obligation to make whole for the damages applies. Ophthalmologists are classified under the no result obligation. In case of fault, “malpractice” is characterized and damages must be paid.

According to data published by the US National Academy of Sciences, from 44 to 98 thousand people die annually due to the country’s medical malpractice, and the financial loss resulting from the problem is approximately US$17 to 29 billion annually.^4^ In developing countries, the chances of medical malpractice is even higher due to the inadequate structure, poor equipment and low investment in operational costs essential in health care.^5,6^

In Brazil there is insufficient data on this subject, but studies indicate that the number of charges for malpractice has grown over the last years.^7^

Therefore, the purpose of this study is to examine the subject of medical claims filed with the São Paulo Court of Appeals in the area of Ophthalmology, including medical civil liability in said cases, in light of the 1990 Consumer Protection Code, the 1988 Federal Constitution and the 2002 New Civil Code. Its consequences on the Brazilian doctrine and case law will be discussed.^7,8^

## OBJECTIVE

To conduct a data survey on the subject of medical claims involving civil liability in Ophthalmology at the São Paulo Court of Appeals.

## METHODS

A retrospective study was carried out based on search of the keyword “ophthalmologist ‘in the São Paulo Court of Appeals site (https://esaj.tjsp.jus.br/cjsg/consultaCompleta.do). No specific date was selected so that it covered all years up to June 1, 2016. In this site “Field”, pecuniary damages, malpractice and pain and suffering; and malpractice were chosen. The following decisions were included: Second Level; Board of Appeals (group of judges of the Second Instance of the Small Civil Claims Court); Appellate decisions (Second Instance Decisions taken by three or more Civil Chamber Appellate Judges), Ratification of Settlement Agreements (when the parties mutually agree on a certain amount or action) and Trial Court Decisions (decisions by the Second Instance, which were not taken in group, but by a single judge).

Only civil liability cases where the defendant was an ophthalmologist were chosen, totaling 65 proceedings.

The following variables were analyzed: (1) type of surgery or clinical diagnosis related the proceeding; (2) defendants – the party that was sued (ophthalmologist; clinic or hospital, and city administration); (3) year in which the claim was assigned to the Court of Appeals; (4) absence or presence of expert evidence; (5) granting or denial of the claim by the lower court.

The data obtained was classified and analyzed in Excel. Of the 65 proceedings initially found,36 were excluded: 11 because they were claims filed only against hospital or clinic or a health establishment, and not directly against a physician; 2 were against the hospital, clinic or health establishment related to the health insurance plan; 1 excluded for repetition; 1 against the Health and Assistance Foundation of the city of Caçapava-FUSAM; 1 against the health insurance plan; 1 excluded because it was filed against a false physician, and 1 because it referred to an ophthalmological procedure not treated by an ophthalmologist. Of the remaining process excluded, 18 proceedings involved medical malpractice in which no ophthalmologist was involved: 5 were filed against emergency physicians, 2 related to procedures conducted by surgeons from other specialties, 2 were non-ophthalmologist physicians, 2 were ear nose and throat specialists, 2 were obstetricians, 1 was a homeopathy specialist, 1 plastic surgeon, 1 neurosurgeon, 1 neurologist, and 1 orthopedic surgeon. Accordingly, the 29 proceedings not excluded were related to civil liability of the ophthalmologist, who appeared as the defendant of the case for alleged medical malpractice, analyzed by the study.

## RESULTS

Of the proceedings analyzed, in 21 cases (72%) the ophthalmologist was the sole defendant in the claim, in 7 (24%) the ophthalmologist was a defendant together with the health organization (either clinic or hospital), and in one case (3.4%) the ophthalmologist was a defendant together with a city administration.

**Figure 1:**
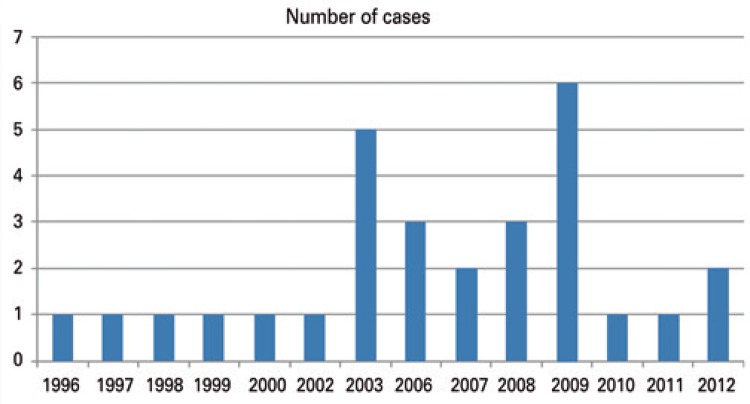
Proceedings between 1996 and 2016. No case found after 2012

A huge growth in the number of cases was noted in the period from 2000 to 2015 (Figure 1). We divided the procedures performed by physicians into two types: clinical and surgical, in which surgical cases prevailed substantially (Table 1). In 28 proceedings (97%) expert evidence was requested in the court records. In the proceeding where an expert evidence was not ordered by the judge, the request for an expert evidence was under analysis.

**Table 1:** Clinical and surgical ophthalmological procedures examined in the proceedings

Clinical case proceedings	n	Surgical case proceedings	n
Contact lenses adjustment	2	Macula hole surgery	1
Neovascular glaucoma	1	Removal of eyelid injury	1
Retinal laser therapy	1	Cataract	10
Epilation for trichiasis	1	Pterygium	2
Glaucoma	2	Refractive surgery	5
Trauma	1	Retinal detachment surgery	1
		Epiretinal membrane surgery	1
Total	8	Total	21

Of the 28 proceedings in which the judge granted the production of an expert evidence; in 4 the expert evidence was not conclusive; in 3 malpractice was found; and in 20, no malpractice was found. Moreover, in 1 case either the decision or the data was pending, as it was related to an old case and the court records had not yet been fully digitalized.

Of the 29 proceedings, 2 had not been tried yet and 21 (72%) were dismissed by the lower court. Of those, all had expert evidence which did not prove the physician’s fault. On the other hand, there were two cases fully granted and two partially granted.

## DISCUSSION

The studies involving analysis of proceedings in Brazilian courts have some limitations. Firstly, case law research conducted refers only to proceedings whose decisions have already been rendered. Furthermore, the mechanisms of court search are limited and some data are lost due to classification mistakes by clerks, for example.

Delay in hearing the cases is also responsible for jeopardizing the analysis of data. Precisely, for that reason our analysis was based on lower court decisions, considering that the decision on appeals filed at the higher court may take up to 2 years to be decided. This is so because the Brazilian legal system affords a number of possible appeals for proceedings filed, and that, despite generating juridical security and consecrating the right to a fair trial may be extremely time-consuming until the decision becomes *res judicata*, that is, it becomes final and unappealable.

Accordingly, taking into account the above considerations, it is possible to see in the period analyzed, the increase in the number of proceedings involving ophthalmologists. Most proceedings were of surgical nature and were related to the anterior chamber of the eye (65%). In four cases (13%), the procedures took place in urgency and emergency situations. The remaining ones were conducted in clinical or elective surgery situations. In 18 cases (approximately 62%), the cause for filing the proceeding was dissatisfaction with the result of the surgical procedure.

The conclusion shows the importance of expert evidence in the court records, where 100% of claims were granted. In the only exception the judge was silent about it.

Accordingly, when conducting the analysis of the cases, it was possible to see that many times experts request a copy of the patient’s records, which shows the large importance of this document as a piece of evidence.^7^

Maia et al.^7^ have analyzed 43 proceedings at the Maranhão Court of Appeals about medical malpractice, and the most common charges pressed were for unintentional bodily injury (51.1%), followed by involuntary manslaughter (37.8%), particularly negligence (49%). In 93%, a copy of patient’s records had been brought to the court records.

In article 94, Civil Code of 2002, it is written that “in bilateral acts, the intentional silence of one of the parties about a fact or quality that the other party has ignored constitutes willful omission, proving that without that the agreement would not have been executed”. This shows the importance of patient being duly clarified before any medical procedure, using plain language.

Several factors are involved with the increase in the number of proceedings for malpractice, such as the population deeper knowledge about their rights, poor working conditions, especially in the government sectors, and media influence. Some of the most important factors in creating this situation are the deterioration of the quality of the doctor-patient relationship and the poor education of physicians during undergraduate and graduate studies.^8,9^ Recognition of the role of medical education in preventing malpractice must be discussed, particularly due to the growing number of medical schools in the country.^10^

To prevent medical malpractice, the most important aspects to be addressed during undergraduate studies are the improvement in the doctor-patient relationship, and communication between doctor, patient and family members, in addition to the encouragement to the proper filling up of medical records.^11^ Legal aspects must also be studied as part of the medical education during undergraduate studies, because according with Decree-Law 4,657, dated September 4, 1942, in its article 3 provides that, “no one is excused from having to abide by the law alleging its ignorance”.^10,12-14^

According to the data published by the American Medical Association, a little more than 42% of all physicians in the United States and 57% of surgeons in different specialties have already been sued. Approximately, 61% of physicians who are 55 years old or older have already been subject to a legal claim.^15^ It is believed that these numbers may be reduced by means of a better and more properly documented communication with the patient. To reduce the gap between patient’s expectations and the results of procedures, a process of standard informed consent may be used, so that patients may fully understand the procedure and possible results. It is necessary to encourage the instrument of knowledge and consent for several medical procedures, currently imposed by the Consumer Protection Code. This instrument of responsibility does not release the physician from being sued but may help their defense, and show the responsibility of the parties involved.

Ophthalmology is not the specialty with the largest number of proceedings, according to the literature. Studdert et al., analyzed data from five US insurance companies and found a very small percentage of injuries proved by physicians. The specialties with highest indexes of proceedings were: gynecology/obstetric, general surgery and internal medicine.^16^

Data obtained from the literature reinforced those found in this study. Among the ophthalmological processes analyzed, the surgical ones are responsible for over 90% of cases.^17^

Medical civil liability has become a subject of major importance due to the increase in the alleged medical malpractice shown by the media and the current trend to health care judicialization. Physicians lack legal education, but need to be informed to practice their profession with tranquility and security.

## CONCLUSION

When researching medical proceedings in the São Paulo Court of Appeals, an increase in the number of ophthalmological proceedings was noted. New studies are called for on this subject in other country regions. It is important to invest in malpractice prevention, and encourage discussions starting during medical undergraduate studies aiming at preparing professionals to be more committed to the medical practice.
